# Recovery with vistula tart cherries following a marathon

**DOI:** 10.1007/s00394-025-03847-y

**Published:** 2026-02-12

**Authors:** Emma Squires, Ian. H. Walshe, Ashleigh Keenan, Oliver Hayman, Ishbel Lomax, Jacob Wood, Rosiered Brownson-Smith, Malachy. P. McHugh, Glyn Howatson

**Affiliations:** 1 Faculty of Health and Life Sciences, Northumbria University, Newcastle, UK; 2 School of Cardiovascular and Metabolic Health, BHF Glasgow Cardiovascular Research Centre, College of Medical, Veterinary, and Life Sciences, University of Glasgow, Glasgow, UK; 3 Translational and Clinical Research Institute, Newcastle University, Newcastle, UK; 4 Nicholas Institute of Sports Medicine and Athletic Trauma, New York, NY USA; 5 Water Research Group, North West University, Potchefstroom, South Africa

**Keywords:** Inflammation, Muscle damage, Nutrition

## Abstract

**Purpose:**

Long-distance running induces marked increases in inflammation and muscle damage. Tart cherries (TC) have become a popular nutritional strategy for exercise recovery, particularly for attenuation of markers associated with muscle damage and inflammation.

Research question.

The aim of this study was to examine the effect of a yet-to-be-explored cultivar (Vistula) of TC on recovery following a marathon.

**Methods:**

Thirty-five recreationally trained marathon runners (mean ± SD age, stature, and mass were 40 ± 10 years old, 176.5 ± 10.2 cm, and 78.8 ± 13.8 kg, respectively) completed an undulating marathon course. Participants were randomised to receive either freeze-dried TC powder or calorie-matched placebo (17 TC, 18 placebo) for 7 days, with the marathon on day 5 of supplementation. Maximal voluntary contractions (MVC), counter movement jumps (CMJ), muscle soreness (DOMS), plasma creatine kinase (CK), and high-sensitive C-reactive protein (hs-CRP) were assessed before, immediately after, and at 24- and 48-h post marathon.

**Results:**

There were significant changes over time for all variables (*p* < 0.001) indicating muscle damage. There were no treatment or interaction effects for MVC, CMJ, DOMS, and CK (*p* > 0.2). However, there was a treatment effect for hs-CRP, where the TC group experienced lower levels of hs-CRP (mean ± SD during recovery: TC 7.9 ± 3.5, placebo 12.5 ± 5.1 mg/L, *p* = 0.031).

**Conclusions:**

The marathon caused changes in muscle damage and inflammation indices. Despite no functional or soreness differences, the TC group showed lower inflammation levels, offering insights into Vistula tart cherries for recovery.

**Supplementary Information:**

The online version contains supplementary material available at 10.1007/s00394-025-03847-y.

## Introduction

It has been long established that bouts of strenuous exercise evoke a stress response that can lead to muscle damage, inflammation and oxidative stress [1, 2]. In such situations, there is mechanical and metabolic stress from the exercise bout, and the initial response is typically referred to as the primary phase of exercise induced muscle damage; EIMD [3]. The damage to skeletal muscle fibres and connective structures results in a loss of force production and pain of the affected muscle. The structural changes are followed by a local inflammatory response, known as the secondary phase, and there is a disturbance of redox balance, which can be observed for days after the exercise bout [4].

A prolonged endurance event, such as a marathon race, is known to cause systemic inflammatory response [5], because of the high metabolic cost over a long duration and the repetitive eccentric contractions. As a result, individuals experience prolonged strength loss, muscle soreness and inflammation [6], therefore any intervention that might help reduce the negative effects will be of benefit. One proposed method is the use of foods that can exert a physiological effect beyond its caloric value. These so-called functional foods can contain compounds such as polyphenols that exhibit anti-inflammatory and anti-oxidative properties [7]. In particular, Montmorency tart cherries (TC) have benefits on muscle function recovery [6, 8–10], muscle pain [8, 11–13] and the inflammatory responses [6, 10, 11, 14–16], which have been attributed to their antioxidant and anti-inflammatory properties [17]. Regarding long-distance running, previous studies have shown that Montmorency TC supplementation can effectively reduce the signs and symptoms associated with the resulting muscle damage. To date, three studies have explored this [6, 11, 13]; all showed TC can positively influence muscle pain/soreness, muscle function, and/or inflammation following the exercise.

While Montmorency TC were used in most previous studies, to date, no study has utilised the European Vistula cultivar of TC to assess its effects following a marathon. It is well-established that different varieties of the same fruit have different composition of phytochemicals [18], which can be influenced by environmental factors [19]. Moreover, powdered forms of TC have been found to contain higher concentrations of anthocyanins than the concentrate equivalent [20], and could be considered a more convenient form of consumption. Therefore, it makes the expectation tenable that consumption of Vistula TC might also exert a similar effect to the Montmorency cultivar. This study aimed to determine whether 7-day supplementation with powdered Vistula TC extract attenuated the negative effects associated with muscle damage following a marathon run. The results of the study provide new information on the application of a powdered European TC and its practical application to aid exercise recovery.

## Methodology

### Participants

Participants were recruited from a pool of runners taking part in the Kielder marathon, Northumberland, UK. Using a previous study that examined the effects of TC on recovery after a marathon race [6], to detect a group difference of ≥ 10% (SD 8%) in the primary outcome variable; MVC, it was estimated that at 0.80 power and 0.05 significance, the minimum number of participants required would be n = 13 per group. Thirty-eight participants were initially recruited and provided written, informed consent. Three withdrew after baseline assessment (7.9% drop out): two due to illness and one who did not want to consume any intervention before the race. Therefore, 35 recreationally active runners were included in the analysis. A summary of their characteristics is presented in Table 1. To assess eligibility, participants completed a health screening questionnaire; those with a known food allergy, smokers, cardiovascular/gastrointestinal/thyroid/renal disease, or musculoskeletal injury, were excluded from participating. The participants were asked to maintain their habitual diet, and to fill in a short food log when they consumed foods/drinks that were typically high in polyphenols, such as fruits & vegetables, fruit juices, nuts and seeds, chocolate and coffee/tea. Participants were asked to refrain from strenuous exercise (other than completing training runs before the marathon) for the duration of the study and to refrain from non-steroidal anti-inflammatory or analgesic drugs, and nutritional supplements such as protein, and vitamins. This study received ethical approval from Northumbria University Ethics Committee (reference number: 1987) and has therefore been performed in accordance with the ethical standards laid down in the 1964 Declaration of Helsinki and its later amendments, this study was also registered with clinicaltrials.gov (NCT06332222).

**Table 1:** Descriptive data of the volunteer Marathon runners in the Vistula tart cherry and placebo groups

Group	Sex (M/F)	Age (years)*	Height (cm)	Mass (kg)	Daily Polyphenol Intake (mg/d)	Predicted Time (h:min:s)	Actual Time (h:min:s)	Highest Weekly Mileage	Longest Training Run (miles)	Previous Marathons
TC	11/6	36 ± 9	178.1 ± 9.7	78.6 ± 14.5	679 ± 241	04:40:00 ± 00:45:02	04:57:30 ± 00:59:47	23.6 ± 8.6	19.2 ± 4.1	3 ± 5
Placebo	12/6	44 ± 9	175.0 ± 10.7	79.0 ± 13.5	710 ± 327	04:29:43 ± 00:38:24	04:48:37 ± 00:47:25	25.5 ± 10.8	23.4 ± 8.1	11 ± 36

Values are mean ± SD; Vistula TC (TC) n = 17, placebo n = 18. * denotes a significant difference between the groups (*p* = 0.01)

### Experimental design

The study design was a parallel arm, repeated measures design that was double blinded, randomized, and placebo controlled. Participants were assigned to one of two groups, to consume either the TC capsules or placebo capsules, matched for caloric content. Participants were required to attend the laboratory for a familiarisation, baseline assessment (which took place 5–6 days before the race), and 24- and 48-h post-marathon. Participants had an assessment of their functional and perceptual measures of recovery, pre-, immediately post-, 24- and 48- h post-marathon. The loading phase for the intervention ingestion commenced four days prior to the marathon and included both the day of the marathon as well as the two following days, totalling seven days.

### Marathon characteristics

The marathon took place at Kielder Water, Northumberland, UK on the 08/10/23, and consisted of an almost entirely off-road course around northern Europe’s largest man-made lake, Kielder Water, with a total ascent of 613 m and total descent 616 m. The environmental conditions on the day of the race were: barometric pressure, 1023 mb; temperature, 16 °C; wind speed, 7 km/h; humidity, 99%. The conditions were wet under foot and there was a mist in the air causing reduced visibility (~ 50 m).

### Treatment conditions and dietary polyphenol estimation

The participants consumed either a placebo or a TC spray-dried extract made from a variety of TC know as ‘Nadwiślanka’ also called Vistula Cherries (extract brand name CherryCraft®, Iprona Lana SpA, South Tyrol, Italy). The intervention was consumed in the form of capsules, twice per day for a period of seven days. The supplementation period was similar to previous studies and a recent meta-analysis [9, 21–26]. The dose increased on the day of the damaging stimuli to increase the bioavailability of phytochemicals when exercise stress and its repercussions were likely elevated; previous bioavailability studies have shown that increasing the dose can elevate anthocyanin and phenolic acids [27–29]. The participants were required to consume a lower dose for the first four days, followed by a higher dose (68.4 and 123.1 mg of total anthocyanins expressed as cyanadin-3-glucoside measured via high-performance liquid chromatography and 339.9 and 611.9 mg of total polyphenols measured via the folin-ciocalteu method [30], respectively) for the remaining three days (day of marathon protocol, 24 and 48 h post). The loading dose was determined by replicating the average reported anthocyanin content from previous studies which used powdered TC products [11, 12, 31]. Then, the higher dose was approximately double, similarly to the papers which have observed some differences in dose–response relationship studies [27, 28]. The chromatogram (supplementary information, Fig. 1) and quantification of anthocyanins (supplementary information, Table 1) in this batch are available in supplementary information. Daily polyphenol intake was estimated using Phenol-Explorer®.

**Fig. 1:**
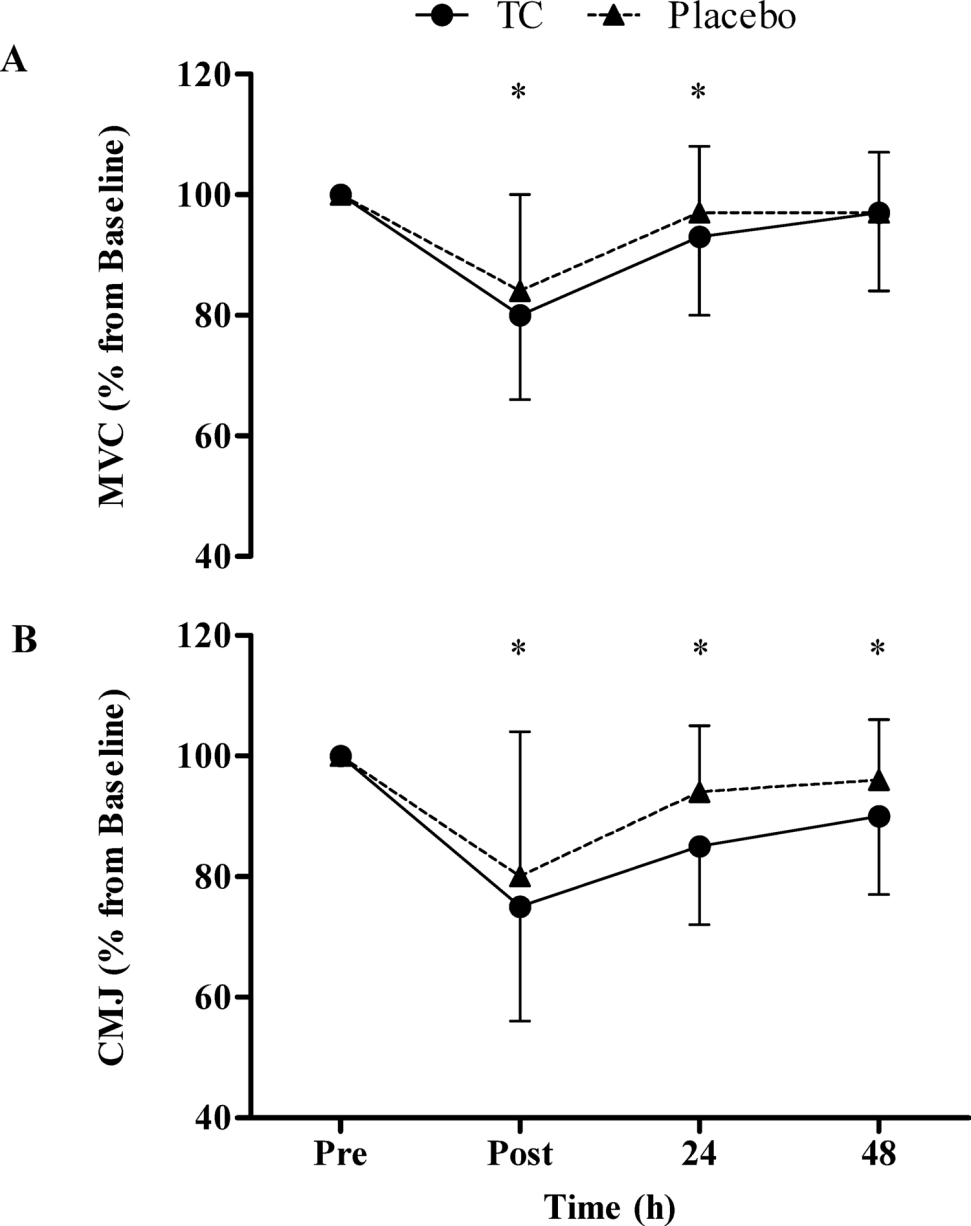
**a** Maximal voluntary contraction (MVC) and **b** counter movement jump (CMJ) percentage change from baseline (pre), immediately post (post), 24- and 48-h post a marathon. Values are mean ± SD; * denotes a significant time effect compared to baseline (*p* ≤ 0.005)

### Functional performance and perceptual variables

After a familiarisation (one week prior to baseline assessments), participants were assessed for their fatigue and recovery via measures of functional performance and perceptual variables at baseline (5–6 days prior to the race), immediately post, 24- and 48-h post- marathon.

#### Maximal voluntary contraction (MVC)

As described previously [6, 32], MVC was measured with a portable strain gauge (MIE Medical Research Ltd., Leeds, UK). All assessments were performed with the participants seated in an upright position. After adjusting the strap to ensure a 90° knee joint angle was attained (verified with a goniometer), participants were instructed to push against the strap with maximal force for a 3 s contraction. The peak value (N) from three maximal contractions (separated by 30 s) was used for analysis. Verbal encouragement was provided for all assessments. The technical error of measurement (TEM) was 27.66 N (1.28%).

#### Counter movement jump (CMJ)

CMJ height was measured using an Optojump system (Microgate, Bolzano, Italy), which calculated jump height (cm) via flight time. When performing the jumps, participants were instructed to keep their hands on their hips throughout the full movement. Participants were required to descend into a squat and jump vertically with maximum effort. Three maximal efforts were performed, separated by 30 s of passive (standing) recovery, and the average value was used for data analysis. The TEM was 0.89 cm (1.19%).

#### Active muscle soreness

Delayed onset muscle soreness (DOMS) of the participants lower limbs was assessed via the use of a 200 mm visual analogue scale (VAS). Participants rated their lower limb soreness after performing one squat (at approximately 90° knee flexion) on a line with the far-left end point representing 'no pain' (0 mm) and the far-right end point representing ‘extremely painful’ (200 mm).

#### Perceptual wellness variables

While seated the participants rated their feelings of soreness (passive), fatigue, energy, sleepiness and readiness to train using a VAS with the far-left end point representing ‘not at all’ (0 mm) and the far-right end point representing ‘extremely’ (200 mm).

### Blood sampling procedure

Venous blood samples were collected from a vein at antecubital fossa on four occasions (pre, immediately post, 24 and 48 h post the marathon). Samples were immediately centrifuged (3000 × g) at 4 °C for 15 min and the supernatant was aspirated into aliquots and then stored at − 80 °C until analysis. Serum was later analysed for CK and hs-CRP using an automated device (Cobas c702, Roche Diagnostics, Switzerland), with repeatability coefficient of variation 0.5%.

### Statistical analysis

All statistical analyses were conducted using SPSS for Windows (SPSS Inc.) and expressed as mean ± standard deviation (SD). A mixed model analysis of variance (ANOVA) with 2 treatment levels (TC versus placebo) and 4 time points (pre, post, 24 and 48 h post exercise) was used to test for differences between the dependent variables. Data analyses for CMJ and MVC were conducted using percentage change from baseline values to account for individual variability. Mauchley’s Test of Sphericity was conducted to test for homogeneity of data and where violations were present; Greenhouse-Geiser adjustments were made. LSD *post-hoc* analysis was used, where necessary, to identify significant time and interaction effects. A significance level of *P* < 0.05 was set prior to all analyses, and a small effect size (ES) was set at ≥ 0.01, medium = 0.06 and large ≤ 0.14, using partial eta squared.

## Results

Participants reported no side effects from the intervention. There were no significant differences in daily polyphenol intake, previous marathon history, weekly mileage, longest single training run, predicted finish time and actual finish time (Table 1), and hence the groups were generally well matched. This was the first marathon for eight participants in the TC group and three in the placebo group. There is a significant difference between the ages in the groups (*p* = 0.01).

MVC (Fig. 1a) showed differences over time (F(_1,3_) = 28.286, ES = 0.462, *p* < 0.001), but no differences between the groups (F(_1,3_) = 0.632, ES = 0.019, *p* = 0.432) and no time x treatment interactions (F(_1,3_) = 0.319, ES = 0.010, *p* = 0.811). Post-hoc analysis revealed statistically significant differences over time in strength from baseline to: immediately post (*p* < 0.001) and 24 h post exercise (*p* = 0.005). Average strength loss (immediately post untill 48 h) was 10% in the TC group vs. 7% in the placebo. CMJ (Fig. 1b) displayed differences over time (F(_1,1.820_) = 27.789, ES = 0.457, *p* < 0.001), but no differences between the groups (F(_1,1.820_) = 1.452, ES = 0.045, *p* = 0.223) and no time x treatment interactions (F(_1,1.820_) = 0.881, ES = 0.026, *p* = 0.411).

A difference over time was demonstrated for active soreness (F_(1,2.231)_ = 101.589, ES = 0.775, *p* =  < 0.001), passive soreness (F_(1,3)_ = 149.790, ES = 0.819, *p* =  < 0.001), fatigue (F_(1,3)_ = 110.736, ES = 0.770, *p* =  < 0.001), energy (F_(1,3)_ = 33.317, ES = 0.502, *p* =  < 0.001) and readiness to train (F_(1,3)_ = 54.871, ES = 0.624, *p* =  < 0.001; Table 2). Sleepiness demonstrated no difference over time (F_(1,3)_ = 2.270, ES = 0.064, *p* = 0.085). All perceptual variables showed no time x treatment interactions (*p* > 0.05) or differences between the groups (*p* > 0.05).

**Table 2:** Indices of muscle soreness and other perceptual variables for the Vistula tart cherry and placebo groups before (pre) and after (immediately post, 24 and 48 h) marathon running

	Pre	Immediately post	24	48
Active soreness (mm)*				
TC	15 ± 22	105 ± 56	67 ± 42	31 ± 28
PLC	17 ± 26	119 ± 39	62 ± 34	38 ± 27
Passive soreness (mm)*				
TC	33 ± 28	137 ± 35	93 ± 36	56 ± 38
PLC	21 ± 14	133 ± 26	86 ± 36	48 ± 25
Fatigue (mm)*				
TC	48 ± 43	147 ± 30	68 ± 31	46 ± 30
PLC	41 ± 35	144 ± 24	74 ± 34	44 ± 33
Energy (mm)*				
TC	128 ± 39	60 ± 44	103 ± 40	118 ± 35
PLC	108 ± 41	55 ± 32	86 ± 22	106 ± 29
*Sleepiness (mm)*				
TC	59 ± 43	75 ± 38	74 ± 47	55 ± 36
PLC	52 ± 42	67 ± 40	72 ± 38	76 ± 45
Readiness to train (mm)*				
TC	133 ± 45	27 ± 33	76 ± 45	119 ± 35
PLC	111 ± 50	39 ± 39	82 ± 40	109 ± 38

Values are mean ± SD. * denotes a significant main effect for time (*p* < 0.001). There were no time x treatment interaction effects or differences between the groups

**Fig. 2:**
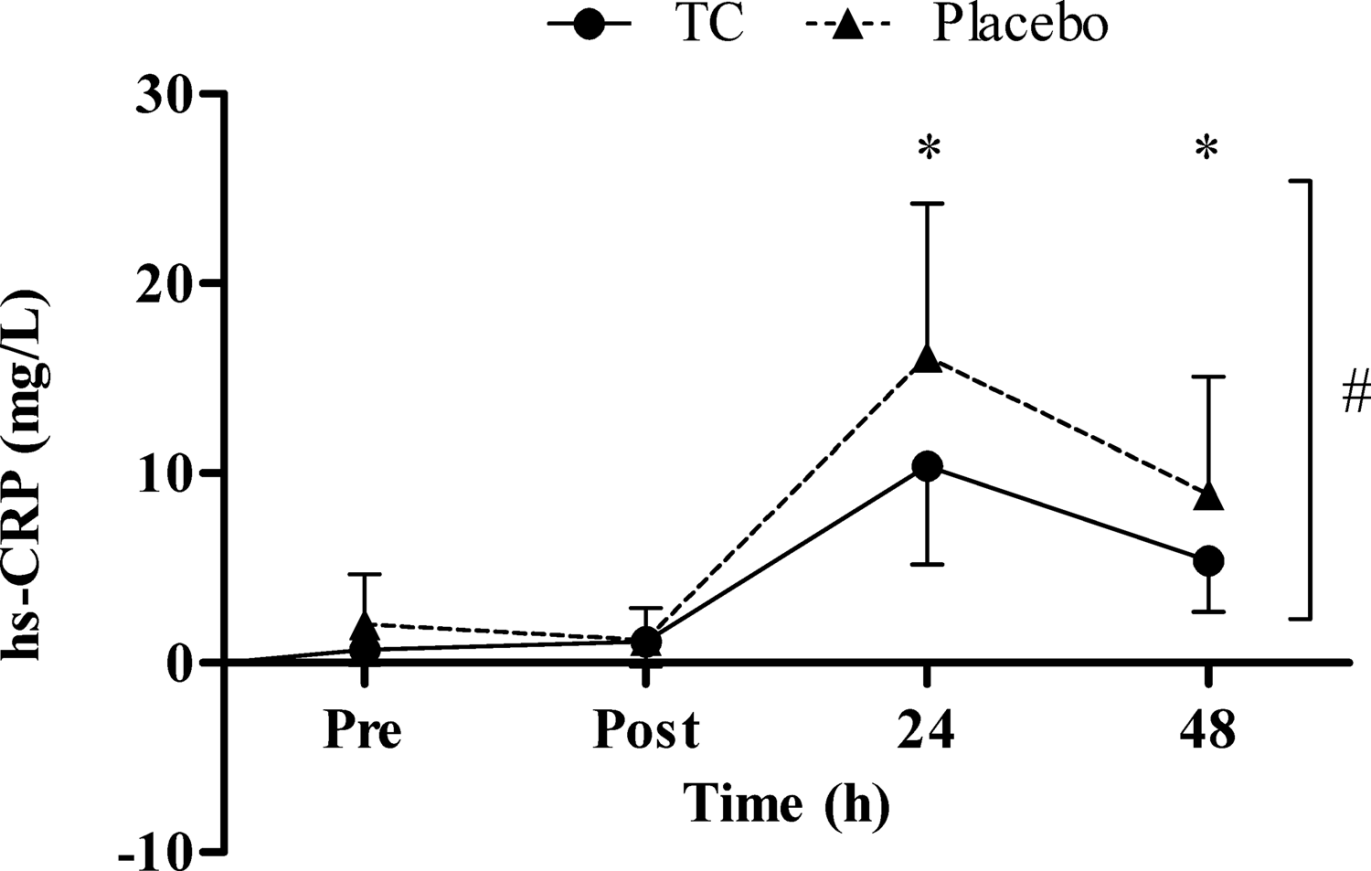
Serum high-sensitive C-reactive protein (hs-CRP) concentration at baseline (pre), immediately post (post), 24- and 48-h post a marathon. Values are mean ± SD; * denotes a significant time effect, where values have significantly increased from baseline (*p* < 0.001), # denotes a significant group difference (*p* = 0.031)

The hs-CRP (mg/L) showed differences between the groups (F_(1,1.360)_ = 5.133, ES = 0.142, *p* = 0.031), differences over time (F_(1,1.360)_ = 108.195, ES = 0.777, *p* < 0.001), and significant time x treatment interactions (F_(1,1.360)_ = 5.109, ES = 0.141, *p* = 0.02; Fig. 2).

CK (U/L) values for the TC and placebo group were 179 ± 108 *vs*. 144 ± 85 at baseline, 445 ± 228 *vs*. 446 ± 299 immediately post, 1419 ± 2081 *vs*. 1448 ± 2142 at 24 h and 1012 ± 1961 *vs*. 941 ± 1447 at 48 h, respectively. CK showed differences over time (F_(1, 1.141)_ = 9.089, ES = 0.227, *p* = 0.003) but no differences between the groups (F_(1, 1.141)_ = 0.003, ES = 0.00, *p* = 0.957) and no time x treatment interactions (F_(1,1.141)_ = 0.014, ES = 0.00, *p* = 0.93).

## Discussion

This is the first study to evaluate the effects of a spray-dried Vistula TC extract on indices of recovery following a marathon. The marathon caused changes in indices of muscle damage and hs-CRP in both groups. Despite there being no evidence of functional or perceptual differences between the groups, the TC group did experience significantly lower levels of hs-CRP following the marathon, compared to the placebo control group.

In the present study, no differences were observed between the TC and placebo groups for either functional measure, MVC and CMJ, after a marathon. There has only been one other study which measured MVC after long-distance running [6], and showed an attenuation in strength loss in the TC group, compared to the placebo. Additionally, after a 109 min cycling trial [14] and adapted version of the Loughborough Intermittent Shuttle Test (LIST ADAPT) completing 6 × 15 min sections [15], similar results were seen, in that Montmorency TC consumption improved the post exercise recovery of MVC. It is feasible that the extent of the damage impacts TC effectiveness. In the present study, immediately post marathon MVC were 80 and 84% of baseline for the TC and placebo groups, respectively. However, Howatson, McHugh [6] reported MVC immediately post marathon as ~ 70% of baseline for both groups, with a ~ 10% greater reduction in force generating capacity compared to the present study. Despite the study being adequately powered (according to our calculations) it is conceivable that the study sample size was still insufficient to detect a group difference. Although, previous work has shown a benefit with very modest changes in strength [14], although in that study cycling was the mode of exercise. A potential explanation could be the training history of the participants, which could influence the extent of muscle damage [33, 34]. In the present study there was an average of 7 ± 25 previous marathons completed, with 11 completing this as their first marathon. This is a far greater range of training status in comparison to the previous study [6], of which the participants had completed 5 ± 7 previous marathons, and only 5 completed it as their first.

This study is the first to investigate the Vistula variety of TC, which is spray-dried and consumed in capsule form. Howatson, McHugh [6] provided the Montmorency variety in a juice blend, which was a mixture of freshly prepared TC juice with commercially available apple juice in a proprietary ratio. Although the anthocyanin content (according to the manufacturer) was greater than previous studies, caution should be taken when interpreting the content of the product. Analytical chemistry methodologies can differ between studies and some previous studies reported the phytochemical content that was derived from previously published studies, not from the batch that was used. Environmental factors can markedly influence the concentrations of phytochemicals in batches of TC [19], ranging from 500 to 2300 mg/L of cyanidin-3-glucoside equivalents in TC juice [35]. Thus, even among TC products of the same cultivar, there will be differences in the concentration of polyphenols and anthocyanins. Hence it is difficult to draw definitive conclusions when comparisons cannot easily be made between studies. When exploring other exercise studies, with different methodologies that used a powdered Montmorency TC product, only one has shown any positive effects [22]; all others reporting no effect with a powdered TC product, compared to the placebo group [11, 12, 36]. However, Hooper, Orange [22] report TC to attenuate strength following EIMD, yet hand-grip strength increased over baseline values, suggesting poor familiarisation with the task. Moreover, given the damage protocol consisted of 60 repetitions of barbell back squats (80% of the participants 1RM), assessing hand-grip strength is not an appropriate measure to assess recovery. Vertical jump performance was also assessed, a more appropriate measure following the damage protocol, although there were no differences between the groups, nor were their performance decrements at any timepoints for that measure. Moreover, there the study was a cross-over design, and a contralateral repeated bout effect (adaptation to the non-exercising limb) might have influenced the extent of damage in the limb tested second bout [37, 38], rendering it difficult to draw conclusions from.

The CMJ data, similarly to the MVC data, contrasted with previous research. Bell, Stevenson [15], Brown, Stevenson and Howatson [39] and Quinlan and Hill [40] demonstrated that TC attenuated the loss in jump height following a damaging bout of exercise. Although the exercise employed is different to the present study, the LIST ADAPT and repeated sprints will likely have induced a combination of mechanical and metabolic stress, in a similar way to marathon running. Despite the observation of jump height improvements in the TC group, there were no differences in MVC recovery between groups [39]. Moreover, eccentric damage to the lower limbs resulted in no differences in the recovery of jump height between the groups [9], yet there were differences between the groups for MVC. There are inconsistent results in previous studies between functional recovery measures; in this case jump height and MVC performance. In the present study, both measures of functional recovery declined following the marathon and the TC group did not experience an accelerated recovery.

Muscle soreness has been proposed to occur via a combination of structural damage to the muscle, disrupted calcium homeostasis, and sensitisation of type III and IV afferent nerve endings from inflammatory cell infiltration [41, 42], although the exact mechanisms remain unclear. In the present study, soreness increased over time, suggesting the marathon was successful in inducing muscle damage. However, the lack of effect Vistula TC had on functional variables, was mirrored in soreness. This concurs with previous research [6, 11, 14] with the exception of one study [13], that showed TC to be effective in reducing DOMS. Kuehl, Perrier [13] only assessed the participants perceptions of pain at baseline, before and after a running race that consisted of distances ranging from 5.6 to 12.4 km. The large range in distance covered suggests that participants are likely to experience a range of muscle damage symptoms. When coupled with the fact that soreness was only assessed immediately after the race, drawing meaningful inference to TC being of real benefit is limited. Despite the criticism of CK as a biomarker of EIMD, it can still be useful to serve as a marker that tissue damage has occurred and the sarcolemma has disruption, but is less useful to determine the magnitude of EIMD [43, 44], unless it is extremely elevated to the point of exertional rhabdomyolysis [45]. In the present study, participants experienced muscle damage as increases in DOMS and CK. Hence, the data concur the consensus of the literature, and it is plausible to conclude that TC products do not attenuate pain (or at least have minimal benefit) following long-distance running.

Soreness (passive), fatigue, energy and readiness to train showed differences over time, but no differences between the groups were observed. Despite the differences between the groups in inflammation, this did not translate to perceptual feelings. This is unsurprising as DOMS was not affected by the consumption of TC, so it seems reasonable to assume other perceptual feelings are not affected. Sleepiness showed no differences over time, nor differences between the groups. Although not significant, 48 h post marathon, there is a 30% difference between the groups in reported perceptions of sleepiness (average for the TC group 55 ± 36 *vs*. placebo group 76 ± 45 mm). Previous research [46, 47], has suggested sleep quality could be influenced by Montmorency TC consumption. Sleep is an important contributing factor to the recovery process [Howatson et al. in press], therefore it seems reasonable to suggest Vistula TC might also improve sleep parameters. Although no differences were observed using a questionnaire, future work could consider using sleep monitoring technology, such as actigraphy.

In agreement with previous studies that observed lower levels of inflammation following endurance exercise with Montmorency TC [6, 11, 14, 16], the present study provided additional evidence that spray-dried Vistula cultivar of TC also attenuated inflammation (hs-CRP) following a marathon. There was a marked reduction in hs-CRP 24 and 48 h post in the TC group, suggesting that the inflammatory response was attenuated. This finding supports the notion that TC supplementation might be more suited to facilitate recovery from exercise that has caused metabolic stress, but little or no effect from mechanically strenuous exercise [21]. One potential explanation for the diminished inflammatory response is that the phytochemicals in TC could reduce the activity of nicotinamide adenine dinucleotide phosphate oxidase, COX, and lipoxygenase, which in turn reduce the formation of ROS [48]. Jung, Kwak and Hwang [49] previously demonstrated that anthocyanins inhibited the COX-1 and COX-2 expression. Another potential explanation could be that TC resulted in a reduction in proteolytic and lipolytic cascades via the COX, prostaglandin, and IL-6 pathways [50, 51]. Prolonged endurance activities, like marathon running, might trigger a systemic inflammatory response, and increased oxidative stress [14]; hence it is plausible the exercise modality and extent of oxidative stress, might amplify TC capacity to alleviate secondary muscle damage. Seemingly, TC supplementation reduced elements of inflammation (as indicated by hs-CRP) but did not allow for greater restoration of muscular performance in the recovery period, which other studies have observed [6, 14, 15].

Potential limitations and weakness of the current study should also be considered. Differences in training status beyond the study inclusion/exclusion criteria could also have been a source of variability in study cohort recruitment. There were no dietary controls in place, which some previous studies have enforced because it allows insight to the potential of polyphenolic compounds. However, this might lead to an overestimation of the intervention effects, and in this case, being a field-based study, no restrictions were in place to increase ecological validity. Lastly, hs-CRP is an immune system protein produced by the liver in response to injury, infection or other inflammatory events [52], hence it is widely accepted as a marker of acute and chronic inflammation. As a result, this measure could be influenced by many factors, and not solely inflammation induced by the marathon. Nevertheless, the major overriding strength of the current study is that this is the first study to be conducted utilising a spray-dried Vistula TC powder.

## Conclusion

This field-based study provides first insights into the use of a spray-dried Vistula TC extract and its effect following a marathon. Although there were no effects on the indices of muscle damage and function, this new intervention was effective in reducing inflammation, was well tolerated and had no adverse effects on participants. Despite the limitations associated with this study, the applied nature provides some external validity that Vistula TC are a promising intervention that could be considered in support of exercise recovery in applied scenarios.

## Supplementary Information

Below is the link to the electronic supplementary material.

Supplementary file1 (DOCX 77 kb)

## Data Availability

The datasets generated during and/or analysed during the current study are available in the FigShare repository, 10.25398/rd.northumbria.25816672.v1.c
