# Supplementary material for: Recovery with vistula tart cherries following a marathon
Source: Eur J Nutr. 2026 Feb 12;65(2):42. doi: 10.1007/s00394-025-03847-y (PMC12901211; doi:10.1007/s00394-025-03847-y)
Supplement: Supplementary file 1 — Supplementary file1 (DOCX 77 kb) [file 394_2025_3847_MOESM1_ESM.docx]

**Supplemental Digital Content (SDC)**


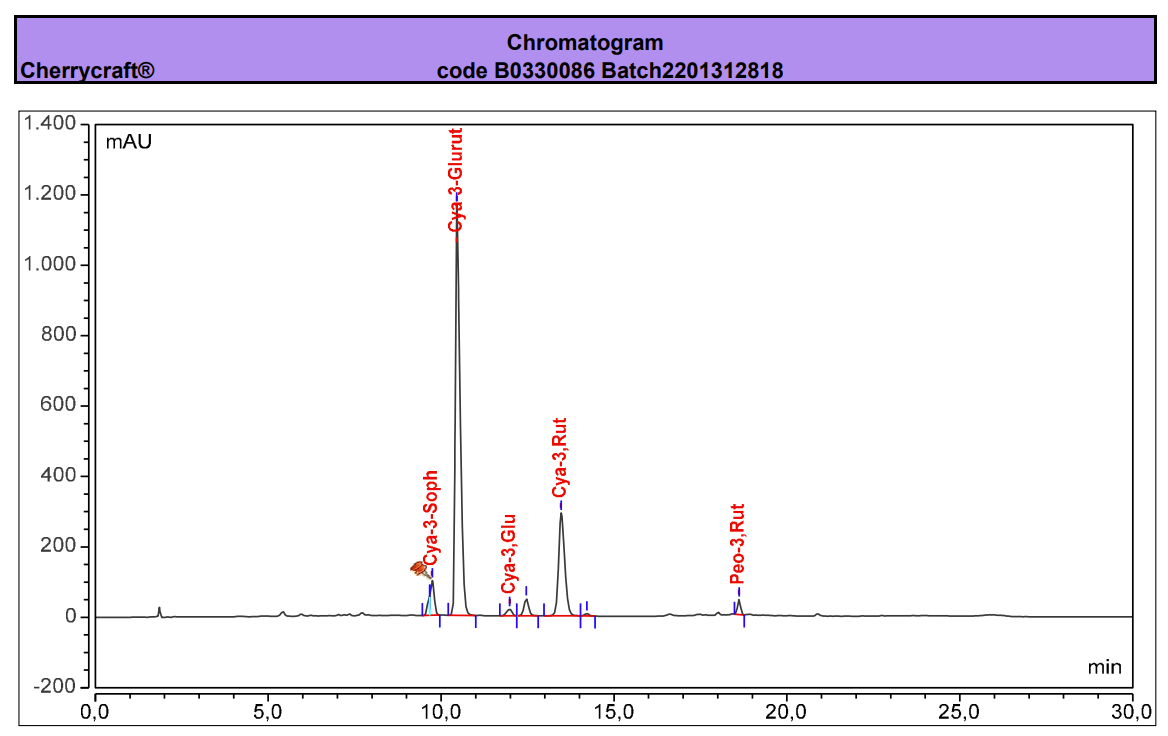


**SDC Fig 1** Chromatogram of CherryCraft^®^ (dried Vistula tart cherry extract) provided in the present study

SDC Table 1. Quantification of peaks identified in CherryCraft^®^ (dried Vistula tart cherry extract) provided in the present study.

| **Peak Name** | **Amount as Cyanidin-3-Glucoside (g/kg)** |
| --- | --- |
| Cyanidin-3-sophoroside (Cya-3-Soph) | 1.01 |
| Cyanidin-3-glucosiderutinoside (Cya-3-Glurut) | 15.42 |
| Cyanidin-3-glucoside (Cya-3-Glu) | 0.28 |
| Cyanidin-3-rutinoside (Cya-3-Rut) | 4.90 |
| Peonidin-3-rutinoside (Peo-3-Rut) | 0.36 |
